# Thermal Effects in Microfluidic Electrokinetic Flows: From Limitation to Design Opportunity

**DOI:** 10.3390/mi17040498

**Published:** 2026-04-20

**Authors:** Tamal Roy

**Affiliations:** Department of Mechanical & Materials Engineering, University of Nebraska-Lincoln, 900 N. 16th Street, Lincoln, NE 68588, USA; troy5@unl.edu

**Keywords:** microfluidics, electrokinetic flows, thermal effects, Joule heating, electrothermal flows, conjugate heat transfer

## Abstract

Microfluidic electrokinetic flows play a central role in applications such as lab-on-a-chip diagnostics, microelectronics cooling, and biomedical sample manipulation. These systems involve intricate heat transfer processes, including Joule heating from ionic currents, temperature-driven flow instabilities, and coupled thermal–fluid interactions, that crucially affect device performance, reliability, and scalability. Current challenges include non-equilibrium charge dynamics, incomplete thermophysical property data for complex fluids, and thermal crosstalk in integrated platforms. This review summarizes the literature published over the past 20 years, with occasional reference to earlier work, covering the fundamental mechanisms of heat generation and dissipation in electrokinetic microflows; analytical, numerical, and experimental approaches for characterizing thermal effects; and discussion on the limitations and application-driven opportunities. It also highlights open questions and future research directions and offers a comprehensive view of design principles and guidelines for developing robust, thermally optimized electrokinetic microfluidic technologies.

## 1. Introduction

**Figure 1:**
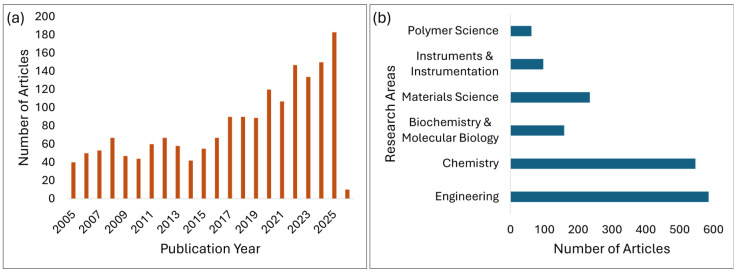
Publication trends on heat transfer in microfluidic electrokinetic flows over the past two decades. (**a**) Bibliographic data from Web of Science depicting yearly publication trends. The search was made using keywords ‘(electrokinetic or electro-osmotic or dielectrophoretic) and (heat transfer or thermal).’ (**b**) Number of published articles on the most significant research areas during the 2005–2026 period.

Heat transfer in microfluidic electrokinetic flows plays a key role in determining the performance, reliability, and integrability of miniaturized systems, including lab-on-chip diagnostics [1,2], electrokinetic pumps [3,4,5], microreactors [6], and emerging microscale cooling concepts [7,8]. In these systems, electric fields are used for transporting fluids and particles, and manipulating species, interfaces, and reactions with high precision. Such geometries are characterized by sub-millimeter dimensions and large surface-to-volume ratios. Under these conditions, the coupling between electric fields, charge transport, and temperature fields becomes non-trivial: even modest voltages can generate significant Joule heating, alter electrolyte properties, and drive complex electrothermal flows that feed back into the electrokinetic transport. As these devices move from proof-of-concept demonstrations to robust, high-throughput, and field-deployable platforms, understanding and controlling heat transfer in electrokinetic microflows has become a key scientific and engineering challenge. There have been numerous studies on heat transfer in microfluidic electrokinetic flows in the past two decades with a monotonic rise in the number of published articles, as summarized in Figure 1a. The most significant area of application includes engineering and chemistry, followed by materials science, biochemistry, instruments and instrumentation, and polymer science (Figure 1b). This growing literature pool indicates the significance of studying the thermal effects in microfluidic electrokinetic flows.

In conventional pressure-driven microchannel flows, heat transfer is analyzed in terms of classical forced convection with well-established correlations for Nusselt number and thermal entrance effects, modified to account for the high surface-area-to-volume ratio and possible rarefaction [9]. By contrast, electrokinetically driven flows are inherently linked to the structure and dynamics of the electric double layer (EDL), ionic conduction, and field-induced body forces within the flow domain. In order to account for these additional effects, the energy equation is solved in conjunction with the Poisson-Nernst-Planck or related electrostatic and species-transport equations, with internal volumetric heat generation due to Joule heating and, in some cases, viscous dissipation [10,11]. Temperature, in turn, affects local viscosity, permittivity, conductivity, and even EDL thickness, making the problem strongly coupled and nonlinear. As a result, thermal effects, which fundamentally reshape the flow, transport, and device response in many electrokinetic microfluidic applications, demand appropriate attention.

The consequences of this coupling are particularly important in bioanalytical and biomedical microfluidic applications, where electrokinetic phenomena facilitate manipulation of biomolecules and cells [12,13,14]. In capillary electrophoresis, isotachophoresis, electro-osmotic pumping, and electrokinetic preconcentration, temperature gradients influence electrophoretic mobilities, buffer pH, band broadening, and resolution [15]. Local overheating can degrade or denature proteins, nucleic acids, and cells, compromising assay integrity and reproducibility. On the other hand, deliberate exploitation of controlled electrothermal flows and non-uniform Joule heating can be used to enhance mixing, accelerate reactions, or achieve rapid, localized heating for lysis or amplification [6]. The balance between unwanted thermal effects and engineered thermal functionality underscores the need for a rigorous understanding that connects electrokinetic driving conditions, geometry, and materials to the resulting heat generation and removal pathways.

Microfluidic electrokinetic flows are also of growing interest in thermal management and energy applications [7,8]. Electro-osmotic and electrokinetic pumps offer compact, valveless, and scalable pumping solutions for microchannel heat sinks and high heat-flux electronics cooling, where control of flow at small scales is advantageous. However, the same electric fields used to generate flow inevitably produce Joule heating within the fluid, altering temperature distributions and fluid properties, and imposing additional heat loads that must be dissipated. In non-linearly shaped microchannels [16], interplay of electrokinetic and thermofluidic phenomena becomes even more complex, with Coriolis and centrifugal effects interacting with electrokinetic body forces and thermal gradients. Future generations of microscale coolers, lab-on-chip power management units, and electrokinetic energy conversion devices must therefore be designed with a deeper understanding of these coupled thermofluidic phenomena.

The technological context motivates a focused review of this area. The rapid expansion of lab-on-chip and point-of-care diagnostic devices has led to increasingly integrated platforms that combine electrokinetic separation, preconcentration, reaction, and detection modules on a single chip. In such integrated systems, thermal crosstalk between modules, substrate heat spreading, and non-uniform power dissipation become critical design considerations. Electrified lab-on-disc platforms, electrokinetically actuated droplet systems, and electrically driven particle and cell manipulation devices similarly rely on controlled thermal environments to maintain biocompatibility and functional performance. At the same time, the aim for higher throughput and faster processing demands stronger electric fields and higher currents, which exacerbate Joule heating and electrothermal effects. Without a comprehensive understanding of heat transfer in these contexts, scaling up such technologies risks unforeseen thermal bottlenecks and reliability issues.

Based on these facts, a dedicated review on heat transfer in microfluidic electrokinetic flows is pertinent to current and future technologies. The aim of this review article is to summarize the underlying physical mechanisms, modeling approaches, and experimental techniques that describe the interplay of electric fields, ionic transport, and micro-scale geometry to produce and dissipate heat. By organizing the literature across fundamental theory, thermal phenomena (Joule heating, electrothermal flows, conjugate heat transfer), and application domains (bioanalytical systems, microelectronic cooling, and particle manipulation), this review aims to outline relevant design principles and highlight rational design strategies. Furthermore, this article identifies the key challenges and research opportunities, such as better characterization of temperature-dependent properties, improved multiphysics models for non-Newtonian fluids, and integrated thermal management schemes tailored to electrokinetic devices. The resulting perspective can guide researchers and engineers in developing more robust and thermally efficient electrokinetic microfluidic technologies that meet the demands of modern diagnostics, electronics, and energy devices.

## 2. Physical Mechanisms and Governing Equations

**Figure 2:**
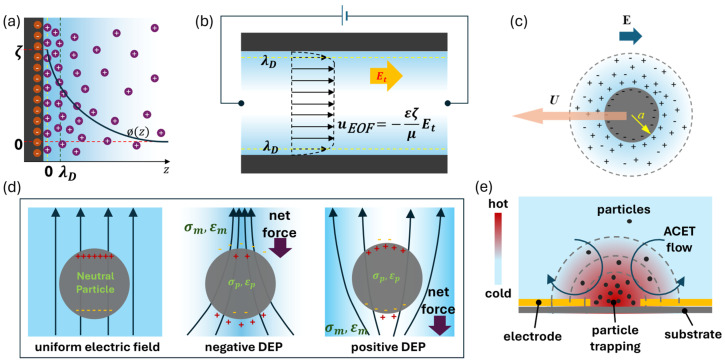
Schematics of fundamental electrokinetic phenomena in a microfluidic environment. (**a**) Concentration of ions near a charged surface, and the definition of the zeta potential. (**b**) Electro-osmotic flow. (**c**) Electrophoresis of a charged particle in an electric field, **E**. (**d**) Dielectrophoresis of a neutral polarizable particle. (**e**) AC electrothermal flow and particle trapping.

Electrokinetic microflows arise from the interaction between applied electric fields and the charged interfaces that develop at solid–liquid boundaries, most prominently in the form of the electric double layer (EDL) at microchannel walls (Figure 2a). When an electric field is applied tangentially to a charged surface, the excess counterions in the EDL experience an electrical body force and drag the surrounding fluid, leading to electro-osmotic flow (EOF, schematically shown in Figure 2b), while charged particles or molecules in the bulk migrate via electrophoresis under the same field (Figure 2c). These mechanisms become significant in geometries with large surface-to-volume ratios, so that interfacial phenomena, rather than pressure gradients alone, can dominate the overall flow behavior and strongly influence transport processes in microfluidic systems.

At the continuum scale, where the characteristic length scale of the domain is significantly larger than the molecule size in a liquid medium [17,18], electrokinetic microflows are often modelled by coupling the Navier–Stokes equations for incompressible flow with Poisson’s equation for the electrostatic potential and Nernst–Planck-type equations for ionic species, forming the Poisson–Nernst–Planck–Navier–Stokes (PNP–NS) framework [17]. The electrostatic potential ϕ satisfies Poisson’s equation:

(1) $$\nabla^{2}\phi =-\frac{\rho_{e}}{\varepsilon },\;$$

where $\rho_{e}$ is the net charge density and ε is the permittivity. For ionic species i, the Nernst–Planck equation yields the conservation of species as

(2) $$\frac{\partial c_{i}}{\partial t}+\nabla \cdot \left(-D_{i}\nabla c_{i}-\mu_{i}c_{i}\nabla \phi +c_{i}u\right)=0,$$

where $c_{i}$ is concentration, $D_{i}$ the diffusion coefficient, $\mu_{i}$ the ionic mobility, and u the fluid velocity. The electrokinetic flows in microchannels inherently possess negligible inertia (due to the micron-scale characteristic length scale, signified by low Reynolds number), and such incompressible flows are described by the continuity and Stokes equations with additional electrical body force, respectively, as:

(3) $$\nabla \cdot u=0,\;\nabla p={\eta \nabla }^{2}u-\rho_{e}\nabla \phi ,\;$$

where ρ is the density, p the pressure, and η the dynamic viscosity. For many microfluidic conditions with thin EDL, the fully resolved PNP system near the wall is replaced by the Helmholtz–Smoluchowski slip condition:

(4) $$u_{\mathrm{EOF}}=-\frac{\varepsilon \zeta }{\eta }E_{t},$$

where ζ is the zeta potential and $E_{t}$ is the tangential electric field, providing an effective electro-osmotic slip velocity at the wall. Such a velocity profile indicates the presence of an effective slip condition at the wall. In micro- and nano-scale electrokinetic flows, this effective slip at the wall often emerges [19]. In general, this slip velocity is characterized by the slip length, defined as $b=u_{t}/{\left(\frac{\partial u_{t}}{\partial n}\right)}_{wall}$, where $u_{t}$ is the tangential velocity of the flow and *n* is the coordinate normal to the wall [20].

Heat transfer in electrokinetic microflows is governed by an energy equation that must be solved in conjunction with the electrostatic and hydrodynamic equations because electric fields and ionic currents introduce volumetric heat generation through Joule heating [21]. In a typical form, the energy equation for the fluid region is:

(5) $$\rho c_{p}\left(\frac{\partial T}{\partial t}+u\cdot \nabla T\right)=k\nabla^{2}T+\Phi_{J}+\Phi_{v},$$

where T is the temperature, $c_{p}$ the specific heat, k the thermal conductivity, $\Phi_{J}$ the Joule heating, and $\Phi_{v}$ the viscous dissipation. Viscous dissipation can be written as:

(6) $$\Phi_{v}=2\eta D:D,\;$$

where D is the rate-of-strain tensor; in many aqueous microflows $\Phi_{v}$ is smaller than $\Phi_{J}$, but it can be significant in high-shear or non-Newtonian regimes.

### 2.1. Joule Heating

When an electric potential is applied across an electrolyte-filled microchannel, ionic currents generate volumetric Joule heating [22,23,24]. For ohmic conduction, Joule heating is expressed as:

(7) $$\Phi_{J}=\sigma {\left|E\right|}^{2},\;$$

with σ the electrical conductivity and E=−∇ϕ the electric field. Because microchannel dimensions are small and thermal resistances in substrates can be large, even moderate voltages can lead to substantial temperature rises and non-uniform temperature fields. These temperature gradients alter local conductivity, permittivity, and viscosity, thereby feeding back on the electric field distribution and electrolyte flow. In electrophoretic and electro-osmotic separation systems, Joule heating can broaden bands, change migration times, and degrade separation efficiency, especially at high field strengths used to increase throughput [25]. However, beyond being a parasitic effect, Joule heating has also been deliberately harnessed as a functional advantage in electrokinetic microfluidic systems, enabling localized, rapid, and energy-efficient temperature control. By exploiting electrically induced heating, researchers have achieved precise thermal regulation for applications such as in situ temperature sensing, biochemical reaction control, and microscale thermal cycling, without the need for embedded heaters or temperature sensors.

Temperature, in turn, feeds back on electrokinetic and flow behavior by modifying thermophysical and electrochemical properties, including viscosity, density, permittivity, ionic mobility, and electrical conductivity. This dependence is often represented through empirical relations such as η(T), σ(T), and ε(T), which enter the momentum, Poisson, and energy equations, and thereby, couple temperature to both flow and electric field distributions. In aqueous symmetric monovalent electrolytes, the Debye length $\lambda_{D}$, expressed by $\lambda_{D}=\sqrt{\frac{\varepsilon RT}{2F^{2}I}},$ where R is the gas constant, F the Faraday’s constant, and I the ionic strength in mM, increases with temperature, affecting EDL thickness, electro-osmotic slip, and screening in narrow channels. This bidirectional coupling between temperature and electrokinetics makes the combined problem inherently nonlinear and can lead to complex phenomena such as thermally induced electro-osmotic instabilities and electrothermal vortices.

Analogous to the electro-osmotic slip length at the wall, a jump or temperature discontinuity at the solid–liquid interface can affect heat transfer in electrokinetic microflows. Such discontinuities occur due to phonon mismatch at the solid–liquid interface and are characterized by the Kapitza resistance [26] (or thermal boundary resistance) and Kapitza length. In electrokinetic microflow, this resistance is characterized by the Kapitza length, expressed as $I_{K}=∆T/{\left(\frac{\partial T}{\partial n}\right)}_{wall}$, where n is the direction normal to the wall. Existence of the Kapitza resistance significantly affects heat dissipation, and the topic was summarized by Chen et al. [27].

An indirect method for applying an electromechanical driving force on the microfluidic flow, which has been explored in the context of microelectronics cooling [5], is through moving suspended microparticles by dielectrophoresis (DEP). A neutral polarizable particle can be manipulated using a spatially non-uniform electric field by DEP. The polarizable particles experience a net force based on the spatial electric field distribution, and electrical properties (permittivity and conductivity) of the particle and the medium [17]. The basic concept is explained in Figure 2d. When the particle is subjected to a spatially non-uniform DC electric field, the net force acting on the particle (of radius *a*) is expressed as:

(8) $$F_{DEP}=2\pi \varepsilon_{m}\frac{\varepsilon_{p}-\varepsilon_{m}}{\varepsilon_{p}+2\varepsilon_{m}}a^{3}\nabla \left[E^{2}\right]\;,\;$$

where $\varepsilon_{m}$ and $\varepsilon_{p}$ are the permittivity of the medium and the particle, respectively. The DEP force, therefore, is proportional to the volume (i.e., *a*^3^) of the particle. When the particle is exposed to a spatially non-uniform AC electric field, the net force on the particle is expressed as:

(9) $$F_{DEP}=2\pi \varepsilon_{m}Re\left[\frac{\varepsilon_{p}\left(\omega \right)-\varepsilon_{m}\left(\omega \right)}{\varepsilon_{p}\left(\omega \right)+2\varepsilon_{m}\left(\omega \right)}\right]a^{3}\nabla \left[E_{rms}^{2}\right]\;,\;$$

where $\varepsilon \left(\omega \right)=\varepsilon -i\frac{\sigma }{\omega }$ and σ is the corresponding conductivity. An AC electric field can be used to apply a net force on the particle, because of the quadratic dependence on the electric field.

### 2.2. Electrothermal and Thermally Induced Electrokinetic Flows

Spatially non-uniform temperature fields create gradients in conductivity and permittivity, which, in the presence of an electric field, generate body forces that drive electrothermal flows [28]. Under an AC field, this electrothermal (AC electrothermal, ACET) effect produces characteristic recirculating vortices that can enhance mixing, pumping, or particle manipulation at the microscale [29,30]. ACET flows are driven by Joule-heating-induced gradients in conductivity and permittivity [31]. The time-averaged electrothermal body force density can be written in terms of conductivity and permittivity gradients, showing its dependence on both electric field strength and temperature sensitivity of material properties. ACET is especially important when AC fields are applied at frequencies from tens of kilohertz to megahertz. Modeling these phenomena requires capturing frequency-dependent behavior of the electric field and the interplay between Coulomb and dielectric components of the electrothermal body force.

ACET models typically combine the following:

- A quasi-electrostatic description of the AC electric field to obtain time-averaged field magnitudes.
- An energy equation with Joule heating term (Equation (7)) to compute temperature fields.
- The Stokes equation (Equation (3)) with an electrothermal body–force density expressed in terms of gradients of conductivity and permittivity [32], as:

(10) $$\langle f_{ET}\rangle =\frac{1}{2}\varepsilon \left[\frac{\alpha -\beta }{1+{\left(\omega \tau \right)}^{2}}\left(\nabla T\cdot E\right)E-\frac{1}{2}\alpha {\left|E\right|}^{2}\nabla T\right]$$

where $\langle f_{ET}\rangle$ is the time-averaged electrothermal body force, ε is the permittivity of the liquid, $\tau =\frac{\varepsilon }{\sigma }$ is the charge relaxation time of the liquid, $\alpha =\frac{1}{\varepsilon }\frac{\partial \varepsilon }{\partial T}$ is the relative change in permittivity with temperature, $\beta =\frac{1}{\sigma }\frac{\partial \sigma }{\partial T}$ is the relative change in conductivity with temperature, and ω is the angular frequency of the AC signal [33]. These electrothermal flows can either be parasitic, disturbing nominal electro-osmotic or electrophoretic transport, or deliberately exploited to improve mixing and reduce diffusion limitations in lab-on-chip systems.

Apart from the externally applied electric field in the electrokinetic microflows, temperature gradient along the channel length can also alter the EDL thickness and thereby induce an electric potential difference and an accompanying fluid flow, known as ‘thermosmosis’ [34]. Thermo-osmotic flow can be viewed as the thermal analogue of electro-osmosis in interfacial transport theory. In charged interfaces, temperature gradients modify the structure and thermodynamic state of the interfacial layer (including the EDL in electrolytes), which generates a net force parallel to the surface and results in an effective slip velocity often termed thermo-osmotic slip. From a continuum perspective, this behavior is captured by a phenomenological slip coefficient that relates tangential fluid velocity at the wall to the imposed temperature gradient, with the coefficient determined by interfacial enthalpy and interaction potentials [35]. When acting on the surface of a particle, the same phenomena generate thermophoretic movement of the particles [36]. Recent theoretical [34], simulation [37], and experimental [36] studies in charged nanochannels and around heated particles have shown that surface charge, nanoconfinement, and wall chemistry strongly modulate thermo-osmotic mobility, and that temperature-induced changes in a double-layer structure can either enhance (at strong EDL overlap, higher ionic concentration, and lower dielectric constant of the solvent) or suppress (at elevated temperatures > 100 °C, where the deterioration of the hydrated structure of the EDL and thermal motion of ions reduce the effective surface charge screening) thermo-osmotic transport.

Dimensionless analysis provides a useful framework for organizing regimes in which different physical effects dominate heat transfer in electrokinetic microflows. A common heat-transfer group is the Péclet number, $\mathrm{Pe}=\frac{UL}{\alpha },$ which compares convective to conductive heat transport, where U is a characteristic velocity, L is a length scale, and $\alpha =k/(\rho c_{p})$ the thermal diffusivity. The Brinkman number, $\mathrm{Br}=\frac{\eta U^{2}}{k\Delta T},$ assesses the role of viscous dissipation relative to conductive transport. Joule heating or electric heating number is defined as $J=\frac{\sigma E^{2}L}{k\Delta T},$ measuring the importance of internal electrical heating. Ratios involving channel height to Debye length, $H/\lambda_{D}$, known as the Debye–Hückel parameter help distinguish between thin and thick EDL limits, and electric Reynolds numbers, ${Re}_{E}=\frac{U\varepsilon }{L\sigma }$, the ratio of charge relaxation to convection time, help distinguish between quasi-steady and dynamically evolving charge distributions. Mapping operating conditions in terms of these dimensionless parameters enables identification of regimes where thermal effects can be treated as perturbations versus those where fully coupled electro-thermo-hydrodynamic modeling is required.

### 2.3. Conjugate Heat Transfer and Boundary Effects

Heat transfer in electrokinetic microflows is inherently conjugate, involving simultaneous conduction in the solid substrate and convection–conduction in the fluid. The effective temperature distribution depends strongly on wall thermal properties, channel aspect ratio, and external cooling conditions, which together determine how Joule heat is removed from the system.

Thermal boundary conditions, such as constant wall temperature, constant heat flux, or mixed conditions, can substantially change local and average Nusselt numbers in electrokinetic flows, compared to purely pressure-driven cases [21]. In some configurations, hydrodynamic and thermal slip (e.g., due to hydrophobic coatings or low conductivity substrates) further modify near-wall temperature gradients and entropy generation, influencing both efficiency and stability of the device [38].

### 2.4. Thermal Effects in Droplet-Based Electrokinetics

Droplet-based electrokinetics leverages electrowetting-on-dielectric (EWOD) and continuous electrowetting (CEW) to manipulate discrete liquid volumes for thermal management and lab-on-chip applications [39]. In EWOD, an applied voltage (typically 20–100 V) reduces droplet contact angle through Maxwell stress at the liquid–solid interface, enabling the precise digital control of droplet position, merging, and splitting. However, the same electric field generates significant Joule heating within the conductive droplet or surrounding electrolyte, with reported temperature rises of 10–50 °C depending on voltage, droplet size (1–50 µL), and frequency [40]. CEW-based continuous droplet trains offer higher throughput for cooling but face similar thermal challenges, as the continuous electrical actuation produces steady volumetric heating that must be balanced against evaporative or convective cooling. Recent studies demonstrate that superhydrophilic surface modifications can reduce thermal contact resistance by improving droplet adhesion to hotspots, cooling surfaces from 172 °C to 100 °C within minutes using multiple 30 µL droplets [41]. Thermal effects thus play a dual role: enabling actuation while limiting maximum voltage and duty cycle. Careful design of dielectric thickness, electrode patterning, and operating frequency (1–10 kHz) is required to maintain temperature rises below critical thresholds for both device reliability and biomolecule stability in diagnostic applications.

### 2.5. Nanofluidic Thermal Transport

When channel dimensions approach or fall below the Debye length ($\lambda_{D}$~1–100 nm), EDL overlap fundamentally alters nanofluidic thermal transport. In the overlapping EDL regime, counterion excess across the channel creates a unipolar conduction environment where ion partitioning and thermal gating effects dominate [42]. Temperature gradients modulate $\lambda_{D}$, permittivity, and viscosity, generating thermo-osmotic slip velocities that either enhance or oppose electro-osmotic flow depending on surface charge and thermal expansion coefficients. Prior studies demonstrate thermal gating ratios exceeding 10:1, where mild heating (ΔT ~10–20 °C) can switch nanochannels from high to low ionic conductance by altering EDL structure [42,43]. Heat transfer is further complicated by modified thermal boundary resistance at solid–liquid interfaces and anisotropic thermal conductivity arising from ion layering. These nonlinear couplings challenge continuum models and necessitate hybrid MD-continuum approaches for predictive design [44]. For energy harvesting and desalination applications, controlled thermal modulation of nanofluidic transport offers unprecedented control over ion selectivity and flux, though practical implementation requires resolving thermal crosstalk between adjacent nanochannels in densely packed arrays [45].

## 3. Characterization of Thermal Effects

Modeling and characterization of heat transfer in electrokinetic microflows rely on coupled multiphysics descriptions and increasingly sophisticated experimental diagnostics. Together, they provide the basis for predictive design of microdevices where electric fields, flow, and temperature interact in complex ways [46,47]. From a theoretical and computational standpoint, the study of heat transfer in electrokinetic microflows has evolved from simplified one-dimensional models to fully coupled, three-dimensional multiphysics simulations that resolve EDL structure, ionic transport, and conjugate heat transfer in the fluid and solid substrates. Early work [48,49,50] assumed isothermal conditions or treated Joule heating as a minor correction; however, more recent research recognizes that strong temperature gradients can cause significant variations in conductivity and permittivity, leading to nonlinear feedback and, in some cases, stabilization of flow instabilities [10,22,24]. Advanced models now include non-Newtonian rheology, such as viscoelastic or Jeffery fluids, which are relevant to biological samples and complex working fluids used in microreactors and energy devices [11,51]. These studies highlight the importance of dimensionless parameters, listed in Section 2, in organizing regimes of behavior and guiding design. The analytical, numerical, and experimental works on the characterization of thermal effects in electrokinetic flows are listed and summarized in Table 1.

**Table 1:** Summary of analytical, numerical, and experimental works conducted on the characterization of thermal effects in electrokinetic flows.

Ref.	Flow/Actuation Type	Geometry	Working Medium	Thermal Effects Considered	Methodology	Main Findings
[21]	EOF, pressure-driven flow	Cylindrical microcannulas	General	Viscous dissipation, Joule heating	Analytical, numerical	Electrokinetic slip and wall wettability accelerate microannular flow, shorten transients, enhance heat transfer, and strongly affect temperature and entropy generation.
[33]	ACET flow	PDMS-glass microchannel with coplanar symmetric electrode	Polystyrene nanoparticles, dispersed in deionized water	ACET	Experimental	ACET flow enables efficient trapping of 100 nm nanoparticles in deionized water, achieving up to 30× concentration enhancement.
[22]	Electrokinetic flows with conductivity gradients	Symmetric T-shaped microchannel	Ferrofluid and water	Joule heating	Experimental, numerical	Joule heating under convective conditions increases electric fields for electrokinetic instability and alters concentration gradient.
[23]	Steady EOF	Two-dimensional straight microchannels	General	Joule heating	Analytical	Joule heating significantly affects electro-osmotic microchannel heat transfer for channel thickness higher than 20 μm. Nusselt number remains independent of source term for constant surface heat flux.
[52]	EOF, pressure-driven flow	Rectangular microchannel	Newtonian liquid	Joule heating	Analytical	Nu increases with the channel aspect ratio, decreases with velocity scale ratio, increases (decreases) with dimensionless Debye–Hückel parameter for surface cooling (heating) for high channel aspect ratio, and increases with higher Joule heating.
[29]	ACET flow	PDMS-glass device with coplanar electrodes	Water	ACET	Experimental, numerical	Electrode design strongly affects microfluidic mixing, capture, and heating; optimal designs vary with voltage, while high voltages favor universal mixing.
[46]	Steady electrokinetic flow	Two-dimensional straight microchannels	Water	Joule heating	Numerical	Modeled electrokinetic flow and heat transfer in microchannels with finite-volume method.
[10]	EOF with time-modulated electric field	Two-dimensional microchannel confined between two infinitely parallel plates	General	Joule heating	Analytical	The effect of Joule heating is limited and can stabilize the flow, through the mechanism due to viscosity variation that produces an out-of-phase contribution to the velocity.
[11]	EOF	Tapered porous microchannel	Jeffery fluid	Viscous dissipation	Analytical	Jeffery fluid exhibits higher axial velocity and pressure effects than Newtonian fluid in electro-osmotic tapered porous flow. Velocity increases with Darcy number but decreases with nonuniformity; electro-osmosis and Brinkman number notably influence temperature and bolus formation.
[51]	EOF	Multimembrane microchannel	Jeffery fluid	General heat source/sink	Analytical, numerical	Nu increases with an increase in the heat source parameter. With an increase in time phase-lag, Nu of the first membrane increases and that of the second membrane remains constant.
[53]	Electro-osmotic entry flow	Straight microchannel with end reservoirs	5 mM phosphate buffer solution	Joule heating	Experimental, numerical	Electrothermal circulations generates at the reservoir–microchannel junction from Joule heating-induced property gradients under amplified electric fields, and the effect of electro-osmotic entry flow increases as AC voltage rises at fixed DC voltage.
[54]	AC electrokinetic flow	Straight microchannel	Water at various electrical conductivities	Joule heating	Experimental, numerical	Demonstrated the N-LIFT, requiring a single dye, to characterize the Joule heating, and the experiments verified the trend that the temperature is proportional to the square of the applied voltage.
[55]	EOF, pressure-driven flow	Two-dimensional microchannel confined between two infinitely parallel plates	General	Joule heating	Analytical	For a given flow rate, Nu computed for a combined pressure–electro-osmotically driven flow can reach up to approximately five times that of the pressure driven flow.
[16]	EOF, pressure-driven flow	Circular microchannel with circumferentially heterogeneous surface properties	General	Joule heating	Analytical, numerical	The average Nu increases as the region with higher heat flux expands and as the zeta potential of the lower-charge region increases, reaching a maximum when both heat flux and zeta potential are uniformly distributed along the channel wall.
[15]	Capillary electrophoresis	Circular capillary	NaCl electrolyte with fluorescein dye as the sample species	Joule heating	Numerical	Joule heating becomes significant at high voltages and large capillary radii. Joule heating raises solution temperature and reduces viscosity and conductivity, accelerating sample migration. Simultaneously, it alters diffusion, electrophoretic mobility, and electro-osmotic velocity profiles, causing band distortion, peak reduction, broadening, and deviation from plug-like transport.
[56]	EOF, pressure-driven flow	Rectangular microchannel heat sink	Water	Joule heating	Numerical	Developed a model for optimization of electro-osmotically assisted pressure-driven microchannel heat sink.
[57]	Electrokinetic flow with longitudinal electric field and transverse magnetic field	Rotating microchannel	General	Viscous dissipation, Joule heating	Analytical, numerical	Hydrodynamic boundary layers shrink, and sharp variations appear in the EDL region at high rotational speeds. The Hall parameter lowers fluid temperature at low Hartmann number (*Ha* = 1) but increases it at higher *Ha*. Nusselt number asymptotics are examined across Brinkman and Joule heating ranges.
[4]	ACET flow with multiphase actuation	Rectangular microchannel with planar electrodes	Phosphate-buffered saline	ACET	Numerical	Electrothermal simulations under single-, 2-, 3-, and 4-phase actuation are reported. Maximum flow rate is obtained for 2-phase; 3-phase and 4-phase show 5% and 11% lower flow rates, respectively, relative to 2-phase.
[1]	AC multiple array electrothermal micropump	Microchannels with square, circular, and triangular cross sections	Phosphate-buffered saline	ACET	Experimental, numerical	An AC MAET micropump with side-wall microelectrode arrays is introduced. Multiple actuation modes are examined numerically and experimentally. Performance is strongly influenced by electrode configuration and phasing, enabling high-flow, high-precision micropumping for conductive fluids in lab-on-chip and drug delivery applications.
[3]	ACET flow	2D rectangular microchannel with electrodes at the bottom	1 μm particles in phosphate-buffered saline	ACET	Experimental, numerical	Parametric effects on ACET pumping in microchannels are examined via simulations and experiments, with optimization using Design Expert and an OpenFOAM solver. Increasing electrode gap 3 times reduces velocity by 40%, increasing small electrode size 4 times reduces velocity by 288%, and increasing channel height raises velocity by 40% before plateau.
[58]	ACET flow	ACET micropump with asymmetric electrodes	KCl solution	ACET	Numerical	A fully coupled ACET model with temperature-dependent properties is developed and compared with a decoupled model. Agreement is obtained at low temperature rise, while discrepancies increase with the temperature. The decoupled model underestimates temperature and velocity. Critical frequencies shift higher with the increase in applied voltage.
[59]	ACET flow with slip velocity on wall	2D rectangular microchannel with electrodes at the bottom	Water	ACET	Numerical	The slip velocity at the channel wall significantly influences the flow field. The presence of slip at the wall increases shear stress, thereby improving pumping efficiency. A larger slip length leads to a higher average pumping velocity. With a glass substrate, the effect of slip velocity became more pronounced.
[60]	EOF, Electrophoresis	Rectangular PDMS microchannels	Sodium bicarbonate buffer solution	Joule heating	Experimental, numerical	Joule heating alters the typical plug-like EOF profile. Simulations show that it accelerates sample transport and distorts the sample band, with effects being more pronounced in PDMS/PDMS channels compared to glass/PDMS channels.
[61]	EOF	Electrokinetic separation chip	20 mM phosphate buffer (pH 7.0) solution	Joule heating, conjugate heat transfer	Experimental, numerical	Experimental (IR thermography) and computational results for electrokinetic chip temperatures are presented. IR measurements provide sub-second resolution with 30 μm spot size. Model accuracy is within ±1 °C. Surface temperatures reflect channel values with an average offset of 1–2 °C under real operating conditions.
[62]	ACET microvortex	Parallel plate electrodes with double-sided tape as spacer	1 μm polystyrene bead suspended in KCl-Tween20 solution	ACET	Experimental, numerical	Transient electrothermal vortices induced by optical heating were investigated using μ-PIV and COMSOL modeling. A 980 nm laser scanned over a colloidal suspension between AC-driven electrodes to generate vortices. Superposed axisymmetric vortices visualized fluid motion in custom rapid electrokinetic patterning (REP) traps, revealing constraints of superposition in dynamic configurations.
[63]	EOF, Electrophoresis	Cylindrical capillary	General	Joule heating	Analytical	Thermal end effects cause sharp temperature drops near capillary ends, leading to localized electric field intensification and pressure gradients for mass continuity. The resulting curved velocity profile and enhanced diffusion increase sample dispersion, while Joule heating accelerates sample transport, thereby shortening electrophoretic analysis time.
[64]	EOF, Electrophoresis	Cylindrical capillary	Tetraborate buffer solutions (pH 9.2)	Joule heating	Experimental	Heat dissipation in glass microdevices is comparable to liquid-cooled fused silica capillaries. Electric field strengths 5–10× higher can be applied before efficiency degrades. Joule heating mainly induces radial temperature gradients, while bulk temperature rise has minor effects; active cooling improves reproducibility and prevents boiling.

### 3.1. Analytical and Reduced-Order Models

Analytical and semi-analytical models remain essential for building physical intuition and for rapid exploration of parameter space in electrokinetic heat-transfer problems. Analytical models are particularly useful for the following:

• Identifying dominant dimensionless groups (e.g., Joule heating number, Brinkman number, electrokinetic Peclet number) and associated regimes.
• Clarifying scaling relations for temperature rise, thermal entrance length, and heat-transfer enhancement or degradation under electrokinetic forcing. However, they are limited when geometries involve roughness, constrictions, complex electrode patterns, or strong property variations with temperature, where numerical approaches become indispensable.

In many cases, these models start from the Poisson–Nernst–Planck–Navier–Stokes–energy (PNP–NS–E) system and invoke thin-EDL, low-Reynolds-number, and Debye–Hückel linearization assumptions to derive tractable solutions for velocity, potential, and temperature fields. For Newtonian electrolytes in simple geometries (e.g., parallel plate or circular microchannels), closed-form or series solutions have been derived for electro-osmotic velocity profiles and temperature fields with volumetric Joule heating, under various thermal boundary conditions such as constant wall temperature or heat flux. These formulations often yield expressions for local and average Nusselt numbers as functions of the electric field strength, Joule heating, and electro-osmotic slip, highlighting how electrokinetic driving modifies classical forced-convection behavior [55].

**Figure 3:**
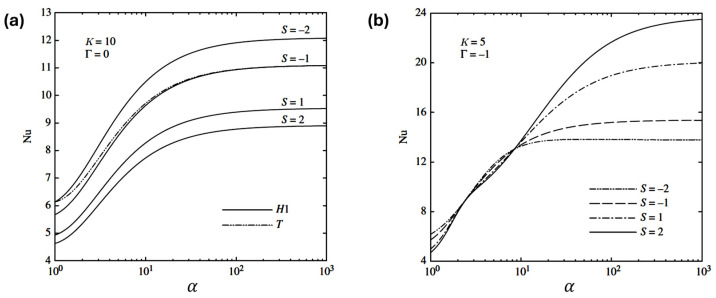
Variation of thermal performance in terms of Nu with varying aspect ratio (α) of the channel for (**a**) different Joule heating numbers (*S*) and (**b**) pressure-opposed flow. Here, *K* indicates the Debye–Hückel parameter and *Γ* represents the velocity scale ratio. Reprinted with permission from [52].

Sadeghi et al. [52] performed a theoretical analysis of combined electro-osmotic and pressure-driven flow in rectangular microchannels for hydrodynamically and thermally fully developed conditions, using Debye–Hückel linearization-based analytical series solutions and constant wall heat flux boundary conditions. The key parameters that influenced the Nusselt number trends were the Debye–Hückel parameter, velocity scale ratio (the ratio of the pressure-driven flow velocity to $u_{\mathrm{EOF}}$), Joule heating parameter, and aspect ratio of the channel (Figure 3). Their results indicate that the Nusselt number increases with channel aspect ratio and decreases with the velocity scale ratio. A higher Debye–Hückel parameter increases the Nusselt number for surface cooling, but for surface heating, it eventually lowers the Nusselt number at a sufficiently large aspect ratio. Higher Joule heating generally decreases the Nusselt number, though under high opposed pressure, it can instead increase.

More recent analytical work has extended these ideas to non-Newtonian and complex fluids relevant to bio- and energy applications. Electro-osmotic and electro-osmotic/pressure-driven heat transfer in Jeffery-type fluids has been studied using perturbation-based schemes, allowing examination of how fluid rheology, porosity, and channel shape affect temperature distributions and Nusselt numbers [11,51]. For instance, electro-osmosis-driven heat transfer in a tapering duct filled with Jeffery fluid and porous medium was analyzed using low-Reynolds-number and long-wavelength approximations, with exact or semi-analytical solutions providing insight into temperature and pressure-gradient fluctuations under different electro-osmotic and geometric parameters. Similar multi-membrane electro-osmotic pumping models with Jeffery fluids show how rhythmic wall motion, buoyancy, and electro-osmosis jointly control flow, heat transfer, and skin friction in vertical microchannels. From these analytical studies, a few general trends can be identified. First, decreasing the Debye–Hückel parameter effectively increases the relative thickness of the electric double layer compared with the channel size, which flattens the velocity profile and systematically reduces the fully developed Nusselt number. Second, as the Joule heating parameter increases, volumetric heat generation raises the bulk fluid temperature and weakens the effective wall-to-fluid driving temperature difference, leading to a monotonic decline in thermal performance as measured by Nusselt number. Together, these results indicate that strong electrokinetic effects and intense electrical heating generally penalize convective heat transfer. The analytical works on the electrohydrodynamic flows and associated heat transfer were summarized recently by Kundu and Saha [38].

A unique approach for handling such problems is entropy generation analysis [65,66]. Entropy generation analysis provides a thermodynamic framework for optimizing electrokinetic microflows by quantifying irreversibilities from Joule heating, viscous dissipation, and finite-rate heat transfer. The total entropy production rate is distributed into thermal conduction ($S_{\mathrm{gen},\Delta T}$), fluid friction ($S_{\mathrm{gen},\eta }$), and mass diffusion ($S_{\mathrm{gen},c}$) contributions, with Joule heating typically dominating in high-field electrokinetic systems. Studies of mixed electro-osmotic pressure-driven flows [65] report that volumetric entropy generation peaks near channel walls where velocity gradients and temperature differences are the largest, with nanoparticles reducing total $S_{\mathrm{gen}}$ by 20–40% through enhanced thermal conductivity despite increased viscous dissipation. For AC electrothermal micropumps, entropy analysis reveals optimal frequencies where flow enhancement balances heating-induced irreversibilities [38]. These thermodynamic metrics guide design by identifying operating regimes where performance gains (higher flow, better heat transfer) exceed thermodynamic costs, particularly valuable for integrated lab-on-chip systems where power budgets are constrained. The approach also highlights counterintuitive results, such as reduced total entropy at higher mass flow ratios due to diminished thermal gradients dominating over friction increases.

### 3.2. Numerical Simulation of Coupled Electro-Thermo-Hydrodynamics

**Figure 4:**
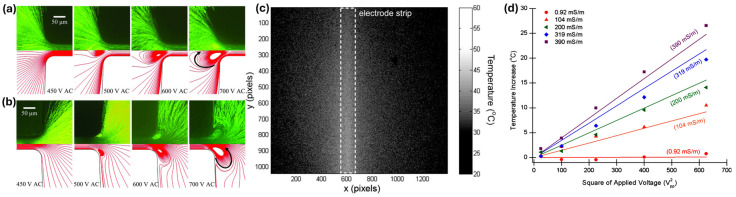
Comparison of experimental and numerically predicted results at the (**a**) inlet and (**b**) outlet of a microchannel. Reprinted with permission from [53]. (**c**) Normalized Laser-Induced Fluorescence Thermometry (N-LIFT) image showing the temperature increase in the medium with conductivity *σ* = 200 mS/m; and (**d**) variation in maximum temperature change with the applied electric potential squared. Reprinted with permission from [54].

High-fidelity numerical simulations form the backbone of contemporary research on heat transfer in electrokinetic microflows, because they can accommodate realistic geometries, conjugate heat transfer, and strong nonlinearity in material properties. Finite volume and finite element methods are typically used to solve coupled equations for fluid flow, electric potential, ionic species, and temperature, subject to appropriate interface and boundary conditions [15,16,46]. Prabhakaran et al. [53] reported the generation of electrothermal fluid circulations at the junctions of the inlet/outlet reservoirs and the microchannel, arising from the interaction of locally amplified electric fields with Joule heating-induced gradients in fluid properties (Figure 4a,b). Their numerical simulation was performed in the COMSOL Multiphysics 5.1, for the continuity and Stokes equations (Equation (3)) with an additional dielectric body force, expressed as $f_{bd}=-0.5E^{2}\nabla \varepsilon$. In addition, temperature-dependent variation in electric conductivity ($\sigma \left(T\right)=\sigma_{\infty }\left[1+\beta \left(T-T_{\infty }\right)\right]$), permittivity ($\varepsilon \left(T\right)=\varepsilon_{\infty }\left[1+\alpha \left(T-T_{\infty }\right)\right]$), and viscosity ($\eta \left(T\right)=2.761\times 10^{-6}exp(1713/T)$) were assumed, where $\sigma_{\infty }$ and $\varepsilon_{\infty }$ are the fluid electric conductivity and permittivity at room temperature $T_{\infty }$, and α and β are the temperature coefficient of the fluid’s electric permittivity and conductivity, respectively. Their results indicate that the intensity of the recirculation increases with the increase in AC voltage, while the DC voltage is fixed. Their model accounted for heat dissipation and electro-osmotic slip at the top and bottom channel walls.

For electrokinetic microchannel heat sinks and mixed electro-osmotic/pressure-driven cooling devices, three-dimensional simulations have been used to optimize channel design and operating conditions [56]. Studies of electrokinetic microchannel heat sinks show that superimposing electro-osmotic flow on pressure-driven flow can reduce thermal resistance and flatten temperature distributions that introduces additional Joule heating and must be reduced for increasing pumping efficiency. Optimization work in these systems often treats geometric parameters (channel width/height, aspect ratio, and electrode placement) and electrical parameters (field strength, zeta potential) as design variables, with objectives such as minimum maximum temperature, minimum thermal resistance, or maximum heat transfer coefficient for a given pressure drop.

Beyond straight channels, numerical frameworks have been developed to study electrokinetic transport and heat transfer in microchannels with random roughness and complex surface microstructure [57,67]. These models reveal that surface roughness can induce local field intensification, recirculation zones, and non-uniform Joule heating, which in turn affect both average and local Nusselt numbers and may promote hotspots or thermal instabilities in practical devices. In rotating or curved electrokinetic microchannels, numerical studies incorporating Coriolis and centrifugal effects show rich thermofluidic behavior, with secondary flows and Dean-like vortices interacting with electro-osmotic and Joule-heating-driven convection.

A few comprehensive reviews on AC electrothermal effect in microfluidics synthesizes these theoretical formulations and compares results from a wide range of micromixers, pumps, and particle manipulation devices [29,30]. These reviews emphasize how geometry, electrode configuration, frequency, and voltage determine flow topology, mixing efficiency, and thermal load and provide practical guidelines for choosing operating conditions that maximize performance while minimizing detrimental heating.

ACET and electrothermal micropumps represent an active area of research for multiphysics simulation. Detailed numerical work has examined ACET microflows under various electrode configurations and phase actuations, solving the coupled electric field, Joule heating, and Navier–Stokes equations to predict flow rates and temperature distributions. For example, a recent study of multiphase AC electrothermal micropumps compared single-, two-, three-, and four-phase driving, showing that multiphase actuation can substantially increase flow rates while maintaining temperature rises within biocompatible limits (<311 K) for biofluids [4]. Similar numerical studies [1,3,58,59] highlight the trade-offs between electrode geometry, actuation frequency, and thermal safety and guide experimental designs that achieve robust pumping without overheating. These models also demonstrate the importance of realistic material properties and conjugate heat transfer in predicting safe operating regimes for organ-on-chip and wearable microfluidic devices.

In addition, numerical analyses of microchannels designed for heat sinks, while not always explicitly electrokinetic, provide valuable conjugate-heat-transfer methodologies and performance metrics (e.g., base-temperature reduction, thermal resistance) that can be adapted to electrokinetically driven microcoolers. Three-dimensional CFD models with conjugate heat transfer in complex-channel geometries define best practices for mesh refinement, residual monitoring, and performance evaluation that are directly applicable to electrokinetic contexts. Across the existing literature, ACET micropumps are typically reported to generate characteristic fluid velocities on the order of 100–1000 µm/s when driven at low voltages (below about 10 V_rms_), moderate electrolyte concentrations (around 10^−2^ M), and high frequencies (from roughly 100 kHz up to the MHz range) to suppress Faradaic bubble formation. Under such conditions, the imposed temperature rise is generally kept modest, with many studies targeting increases of no more than about 5 °C to preserve the viability of biological samples in Joule-heated ACET systems. When substantially higher voltages are applied, however, local overheating can become severe, with reported electrode–interface temperatures rising by several tens of degrees, sometimes approaching 50 °C above ambient.

### 3.3. Experimental Diagnostics for Temperature and Flow Fields

Experimentally, probing temperature and velocity fields in electrokinetic microflows presents unique challenges due to small length scales, fast time scales, and the presence of electric fields that can interfere with traditional measurement techniques. Noninvasive temperature diagnostics, such as fluorescence-based thermometry [60,68], thermochromic liquid crystals [69,70], and infrared thermography [61], have been adapted to microfluidic platforms to map temperature rises due to Joule heating and to quantify heat transfer coefficients. Micro-particle image velocimetry (micro-PIV) and related flow visualization tools allow characterization of electro-osmotic and electrothermal flow structures, including recirculation, vortices, and slip-flow effects at microchannel walls [62]. The combination of high-resolution experimental measurements with detailed simulations offers a path toward validated models and predictive design frameworks for thermally robust electrokinetic devices.

Experimental characterization of heat transfer in electrokinetic microflows hinges on the ability to measure temperature and velocity fields with high spatial and temporal resolution, without perturbing the electric field or flow. Traditional macroscale techniques are often inadequate; so, specialized optical and microfabricated sensors are widely employed. For temperature measurements, fluorescence-based thermometry using temperature-sensitive dyes is one of the most common approaches. These dyes exhibit intensity or lifetime changes with temperature, enabling mapping of temperature fields within microchannels under Joule heating or ACET actuation. Infrared (IR) thermography has been used to measure surface temperatures of microchips, particularly in glass or polymer devices where IR transparency allows approximate reconstruction of fluid temperatures. Williams et al. [54] investigated thermal effects originating from the interaction of AC electric fields and water at various electrical conductivities. They employed normalized laser-induced fluorescence thermometry (N-LIFT) to measure the changes in temperature, where Rhodamine B (RhB) was used as the temperature-sensitive dye (Figure 4c,d). Thermochromic liquid crystals and ionic liquids have also been applied in some configurations, especially for calibration and for monitoring average temperature rises [2,69,70].

On the flow side, micro-particle image velocimetry (micro-PIV) and related techniques are standard for visualizing electro-osmotic, electrophoretic, and electrothermal flow structures. Seed particles are chosen to minimize electrophoretic mobility or to match the fluid’s electrokinetic behavior, reducing bias in velocity measurements, while illumination and imaging are adapted for confined geometries and strong electric fields. Micro-PIV has been crucial in revealing recirculation patterns, vortex formation, and slip-flow-induced modifications to velocity profiles in electrokinetic microchannels and ACET micromixers.

Several studies combine temperature and velocity diagnostics to fully characterize coupled electro-thermo-hydrodynamics. In ACET micropumps, simultaneous mapping of flow and temperature has confirmed that multiphase actuation can increase flow without exceeding safe temperature thresholds, validating predictions from numerical models. In electrokinetic separation systems, spatially resolved temperature measurements have quantified Joule heating effects on separation efficiency and confirmed the need for careful control of buffer conductivity and channel cooling.

## 4. Application-Driven Perspectives

Electrokinetic microflows with coupled heat transfer find diverse applications across biomedical diagnostics, thermal management, and advanced manipulation platforms for cells and particles, where electric fields enable precise control at microscales but introduce thermal challenges that must be managed for practical deployment. This section summarizes key application areas, highlighting how thermal phenomena influence performance and design strategies that leverage or mitigate them. The relevant works on the application of thermal effects in electrokinetic microflows are listed and summarized in Table 2.

**Table 2:** Summary of the works on application of thermal effects in electrokinetic microflows.

Ref.	Application	Materials and Microfluidic Device	Mechanism	Methodology	Main Findings
[71]	Localized micro/nano-electroporation	FAM-labeled oligonucleotides and GFP plasmids as cargos, in 1× PBS buffer. Nano- and microchannel array	Joule heating	Experimental, numerical	Demonstrated coupling among electric field, Joule heating, electro-osmosis (EO), and electrophoresis (EP) in micro/nano-electroporation (MEP/NEP) across varying channel sizes. Single-cell electrokinetic behaviors are analyzed using a microfluidic biochip. Joule heating induces bubble formation, driven by EO toward the cargo side. Increased voltage intensifies EO, reducing cargo delivery efficiency, especially for low-mobility plasmid DNA. An optimal electroporation zone is defined to minimize bubble generation and excessive EO effects.
[72]	Particle manipulation with ACET flow	Polystyrene particles dispersed in KCl. Parallel plate electrodes with double-sided tape as spacer	ACET	Experimental, numerical	An electrokinetic technique is developed to continuously and energy-efficiently manipulate colloidal particles using intrinsic Joule heating. Alternating current electrothermal flows, driven by non-uniform electric and thermal fields, generate strong vortices that concentrate particles at localized hotspots. By tuning hotspot geometry, diverse particle aggregation patterns are formed under low power conditions.
[2]	General microfluidic flow with parallel heating channels carrying ionic liquids for in situ temperature monitoring	Ionic liquids BMIM Imide and BMIM PF6. Meandering PDMS microfluidic channel with co-running heating channel	Joule heating	Experimental	Microfluidic devices enabling localized heating of microchannels were designed, fabricated, and tested. Achieved intra-channel temperature control within ±0.2 °C. Ionic liquids in adjacent channels are Joule-heated with AC current, allowing direct and convenient assessment of internal temperatures.
[6]	Controlling polymerase chain reaction (PCR) thermal cycling in a microchannel using Joule heating	PCR mixture of DNA template, GeneAmp^®^ 1 × PCR Gold Buffer, 3.0 mM MgCl2, 200 μM dGTP, dCTP, dTTP, and dATP (each), 0.25 μM each primer, and 0.1 μM probe. Rectangular microfluidic PCR chip	Joule heating	Experimental, numerical	A Joule heating-based method for PCR thermal cycling in a PDMS microchannel was developed, eliminating external heaters. Internal heating from current flow enabled controlled cycling at only 1.3 W power consumption. Numerical simulations guided parameter selection, and a two-temperature TaqMan real-time PCR successfully amplified the *E. coli* O157:H7 stx1 DNA fragment.
[73]	Temperature control during capillary electrophoresis (CE) separations for spaceflight applications	Mixture of inorganic cations and amino acids using 5 M acetic acid as background electrolytes. Capillary wrapped in a “figure-of-eight” profile	Joule heating	Experimental, numerical	A solid-state capillary temperature control device was developed for integration into in situ instruments. Two prototypes, a thermal mass model (TMM) and a functional model (FM), were tested. The TMM validated gradient minimization and model reliability, while the FM achieved CE analytical performance under active control and thermal-vacuum conditions.
[74]	Cascade EOF micropump for chip cooling	Infinite parallel plates	Forced convection by EOF	Analytical, numerical	A 3D-printed ABS cartridge with inner copper foil (0.2 mm thick) was designed to enhance the capillary electrophoresis–mass spectrometer interface by directing thermostated airflow for efficient Joule heat dissipation along the silica capillary. The cartridge supports up to two capacitively coupled contactless conductivity detectors (C^4^Ds). For monoethyl carbonate separation, peak area reduced by 21% of the original, confirming superior thermal management.
[7]	Active cooling microchannel heat sink device using EOF	0.4 mM borax buffer. Straight rectangular microchannels etched in silicon	Forced convection by EOF	Experimental, numerical	An EOF-driven setup achieved 82 μL/min at 400 V, eliminating high-pressure pumping. Cooling fluid temperature rises of 9.6 °C, 29.9 °C, 54.3 °C, and 80.1 °C occurred for 0.4 W, 1.2 W, 2.1 W, and 4 W, respectively; substrate temperature remained below 80.5 °C. Nu reached a maximum of 5.48 at the channel entrance, 4.56 for the rest of the channel, at 4 W, ~10% higher than pressure-driven flow.
[5]	DEP-based micropump for electronics thermal management	2.9 μm polystyrene particles dispersed in water. Three-phase planer microelectrode array, for generating travelling-wave DEP.	Forced convection by travelling wave DEP	Experimental, numerical	A microfluidic pumping method based on traveling-wave dielectrophoresis (twDEP) of microparticles was developed. Particle motion in non-uniform electric fields generates viscous drag, driving fluid flow. Numerical simulations and μ-PIV experiments quantified and validated the induced flow field. The twDEP micropumping concept shows strong potential for chip-integrated thermal management applications.
[14]	Wearable sensor patch for biofluid monitoring	Microfluidic sweat collection in textile, spray-coated with M-Xene. Laser-engraved PDMS-based microfluidic device.	Joule heating for inducing sweat and subsequent rapid sweat uptake by graphene-based sensors for biomarker analysis	Experimental	A sensor patch was developed for Joule-heating-induced sweating and comfortable biofluid monitoring. Ti_3_C_2_T_x_ nanosheets impart high electrical conductivity to silk fabrics, enabling an efficient low-voltage electrothermal platform. The biosensor triggers noticeable sweating within 5 min via mild thermal stimulation, permitting real-time monitoring of Na^+^, K^+^, pH, and uric acid for metabolic syndrome assessment.

### 4.1. Bioanalytical and Lab-on-Chip Systems

Electrokinetic techniques such as capillary electrophoresis (CE), isotachophoresis (ITP), electro-osmotic pumping (EOP), and field-amplified sample stacking are cornerstones of microfluidic bioanalysis, offering high resolution, minimal sample volumes, and integration potential. However, Joule heating from high electric fields required for fast separations can cause temperature gradients that alter ionic mobilities, buffer conductivity, and sample integrity, leading to band broadening and reduced efficiency [63,64,75,76,77,78].

**Figure 5:**
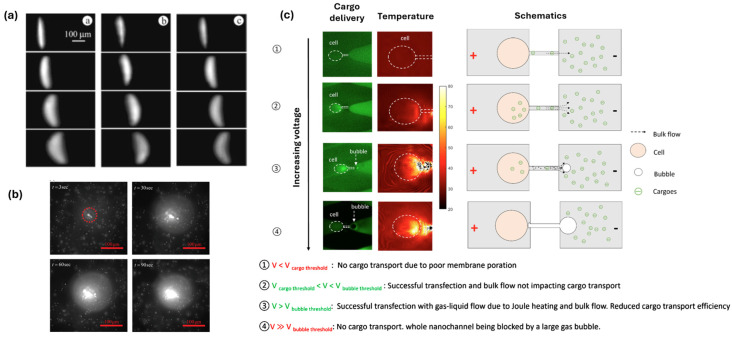
Effects of Joule heating on electrokinetic manipulation of particles and cells. (**a**) Joule heating effect on electro-osmotic flows. Reprinted with permission from [75]. (**b**) Experimentally observed particle aggregation induced by Joule heating. Reprinted with permission from [72]. (**c**) Effect of Joule heating in cellular micro- and nano-electroporation. Reprinted with permission from [71].

In CE and ITP microchips, temperature control is achieved through low-conductivity buffers, tapered channels to reduce field strength, and active cooling via substrate integration or external heat sinks. Studies show that even modest temperature rises (<5–10 °C) can significantly affect resolution in protein and DNA separations, underscoring the need for conjugate heat-transfer models to predict and optimize chip designs [75]. Electrokinetic preconcentration exploits nonlinear charge transport to achieve >10^4^-fold enrichment, but temperature gradients from local heating can induce vortex flows that limit stacking stability [79]. A detailed discussion can be found in the review article by Cetin and Li [75] where the dispersion in electro-osmotic flow due to Joule heating (Figure 5a) is addressed.

On the other hand, previous studies demonstrate that Joule heating can be exploited as a precise and efficient thermal control mechanism in electrokinetically driven microfluidic devices. Kunti et al. [72] demonstrated an AC electrokinetic technique based on non-uniform Joule heating for particle aggregation and pattern formation of particle groups (Figure 5b). The aggregation originated from the coupling between electric and thermal fields, which generates toroidal vortex motion. As a result, the surrounding fluid approaches a targeted hotspot with the particles to form a cluster. De Mello et al. [2] showed that AC-induced Joule heating of ionic liquids enables accurate, contactless temperature control and measurement via conductivity–temperature relationships, achieving stability within ±0.27 °C. Building on this concept, Hu et al. [6] applied Joule heating to microfluidic PCR, electronically controlling denaturation and annealing temperatures through programmed current modulation, enabling DNA amplification with low power consumption (~1.3 W).

Pan et al. [71] performed a combined experimental and numerical study of the effect of electrokinetic phenomena on nano- and micro-electroporation at the single cell level (Figure 5c). Their results indicated the existence of a threshold voltage for bubble formation, which reduced cargo delivery efficiency due to Joule heating and subsequent bubble formation. With increased voltage, both bubble formation and intense bulk flow reduced the cargo transport efficiency.

Electro-osmotic pumping (EOP) provides valveless, pulsation-free flow control in lab-on-chip systems for sample handling, mixing, and gradient generation. Thermal management in EOP is critical because the power dissipation scales with flow rate, potentially leading to thermal runaway or bubble formation in high-pressure applications like micro-HPLC [71,80]. Designs incorporating multiple parallel channels or hybrid pressure–electro-osmotic pumping have been shown to reduce thermal loads while maintaining flow uniformity [25].

**Figure 6:**
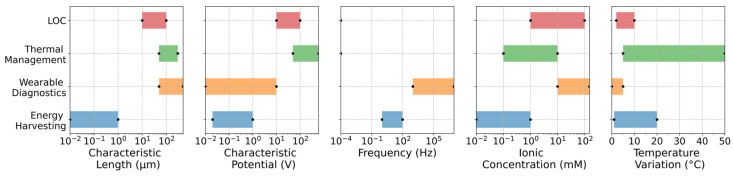
Ranges of key parameters (characteristic length, potential, frequency, ionic concentration, and characteristic temperature variation) for four major applications involving thermal effects in electrokinetic microflows.

Lab-on-disc (LOD) platforms use centrifugal forces for pumping, often augmented by electrokinetics for precise valving, metering, and manipulation. Electro-osmotic flow in rotating microchannels introduces thermofluidic characteristics influenced by Coriolis, centrifugal, and electrokinetic body forces, with Joule heating adding radial and azimuthal temperature gradients that affect assay kinetics and biomolecule stability. A comprehensive review of electrified lab-on-disc systems [81] highlights how integrated electrodes enable electrokinetic preconcentration, lysis, and hybridization on spinning discs, but thermal crosstalk between zones necessitates careful electrode placement and low-conductivity buffers. Numerical models incorporating conjugate heat transfer predict that substrate materials with high thermal diffusivity (e.g., glass or ceramic) are preferable to polymers for LOD applications involving electrokinetics. Recent reviews on electrokinetics in microfluidics emphasize the role of temperature-aware design in scaling bioanalytical platforms for point-of-care diagnostics, where portability limits active cooling options. For instance, polymer microchips with high thermal conductivity fillers mitigate Joule heating, enabling field-deployable CE systems for pathogen detection and biomarker analysis [73,74]. The range of key parameters, including characteristic length, applied potential, ionic concentration of the medium, and characteristic temperature variation, drawn from the existing literature, is summarized in Figure 6.

### 4.2. Microelectronic Cooling and Thermal Management

Electrokinetic microchannel heat sinks leverage EOF’s plug-like velocity profile and low hydraulic diameter to enhance convective cooling in high-heat-flux electronics, such as CPUs, LEDs, and power devices. Unlike pressure-driven flows, electro-osmotic pumping avoids large pressure drops and moving parts, but introduces Joule heating that adds to the electronic heat load and alters fluid properties [7,8]. In another work, traveling-wave dielectrophoresis-based microfluidic pumping was used for thermal management, with extension to nanofluids, where nanoparticles enhanced heat transfer while enabling controlled flow in high-viscosity microfluidic cooling systems [5].

Numerical and experimental studies of electrokinetic microchannel heat sinks demonstrate that optimal performance requires balancing electro-osmotic enhancement of convection against Joule heating penalties. For example, analysis and optimization of electrokinetic microchannel heat sinks show that a hybrid electro-osmotic–pressure-driven configuration can achieve up to 20–30% reduction in thermal resistance compared to pure pressure-driven flows, provided the applied field is tuned to minimize net heating [56]. Key design parameters include channel aspect ratio, zeta potential, electrolyte conductivity, and substrate thermal conductivity, with simulations revealing flattened temperature profiles and higher Nusselt numbers under optimal electrokinetic operation.

In transient pressure-driven electrokinetic slip flows with heat transfer, analytical models predict that thermal entrance effects and Joule heating significantly modify both flow development and temperature profiles, necessitating dynamic control strategies for transient electronic loads [21]. Rotating electrokinetic microchannels for centrifugal microfluidics further complicate thermofluidics, with Coriolis forces interacting with EOF and thermal gradients to produce secondary flows that can either enhance or degrade cooling uniformity [57].

In general, reported systems typically handle average heat fluxes above 150 W/cm^2^. These devices can also achieve very low thermal resistance, with values around 1 °C/W per chip, making them attractive for localized spot cooling. Compared with conventional air cooling, they provide much better compactness, reliability, and noise-free operation because EO pumps have no moving parts. Among various electrokinetic cooling strategies, electro-osmotic-pumped microchannels often rely on electric fields above 100 V to drive the coolant through the channels, while ACET-based devices typically use approximately 10 Vrms. However, Joule heating remains a major constraint, since the same fields that drive flow also generate heat. As a result, design must ensure that the cooling benefit clearly exceeds the electrical heating penalty, especially in 3D stacked chips and high-power-density microprocessors.

Emerging applications include electrokinetic cooling for high-power-density microelectronics and photonics, where nanofluid electrolytes with tailored thermal and electrical properties promise further performance gains. Experimental validation using IR thermography and micro-PIV confirms that electrokinetic enhancement is viable for heat fluxes > 100 W/cm^2^, provided thermal management accounts for coupled Joule and electronic heating [82,83,84]. A summary of the key parameter ranges from the existing literature pool for such applications is provided in Figure 6.

### 4.3. Particle and Cell Manipulation

Electrically driven particle manipulation in microfluidics relies on electrophoresis, dielectrophoresis (DEP), and ACET for sorting, trapping, and assembly, where temperature control is vital for preserving cell viability and functionality. Recent work on microfluidic-based electrically driven particle manipulation reviews how ACET and DEP vortices can achieve rapid focusing and separation, but excessive heating (>42 °C) can damage cells or alter particle surface properties [12].

In ACET-enhanced micromixers and separators, experimental studies using micro-PIV and fluorescence thermometry validate optimal frequency range (100 kHz–1 MHz) to maximize flow while keeping ΔT < 5 °C. This enables label-free cell sorting and nanoparticle assembly. For bioelectrical manipulation like electroporation, localized Joule heating must be confined to avoid nonspecific effects, with simulations guiding electrode geometries for uniform fields and minimal thermal spread [30].

### 4.4. Wearable Diagnostics and Emerging Platforms

Portable and wearable diagnostics increasingly incorporate electrokinetic elements for sweat analysis [85,86], continuous glucose monitoring [87], and biomarker level detection [13]. Thermal management here is constrained by size and power; so, passive strategies like low-conductivity electrolytes and high-κ substrates dominate. Electrokinetic preconcentration in wearable patches enhances sensitivity, but ambient temperature variations and body heat add to Joule heating, requiring robust models for reliable performance. However, in some situations, Joule heating was used for enhanced sweat extraction, thereby negating the need for strenuous exercise, or administration of stimulating agonists [14].

Emerging platforms like electrokinetic energy harvesters and microreactors also benefit from thermal insights. In streaming potential-based generators, temperature gradients affect zeta potential and output power, while in electrokinetic microreactors, controlled heating accelerates reactions without hotspots [45,88]. Figure 6 summarizes the key parameter ranges from the existing literature pool in wearable diagnostics and energy harvesting applications.

### 4.5. Cross-Cutting Design Considerations

Across applications, common thermal management strategies include the following:

- Buffer and electrolyte optimization: Low ionic strength to reduce Joule heating, with temperature-stable properties.
- Geometry and electrode design: Tapered channels, interdigitated electrodes, and multiphase ACET to redistribute heating and flows.
- Material selection: High thermal conductivity substrates (e.g., silicon, diamond-like carbon) and coatings for thermal slip.
- Hybrid actuation: Combining electrokinetics with pressure, acoustics, or magnetohydrodynamics to offload pumping while minimizing electrical heating.

Performance metrics such as thermal resistance, coefficient of performance (cooling power per Joule heat input), and biocompatibility temperature limits guide optimization, with recent studies showing electrokinetic systems can outperform conventional microfluidics when thermally optimized. Future integration with ML-based design and real-time thermal feedback promises even greater advances.

## 5. Open Questions and Future Directions

Despite considerable progress, several gaps and open questions are still existing, which makes this topic timely for a critical review. Many existing models still rely on simplified assumptions of EDL equilibrium, linear property variations with temperature, and idealized boundary conditions, which may not hold true under strong fields, large temperature gradients, or nano-confinement where Debye length approaches channel dimensions. Non-equilibrium EDL dynamics, including finite ionic relaxation times and ion crowding, further complicate charge transport and Joule heating predictions, particularly in transient or high-frequency AC electrothermal flows. Thermophysical properties of complex biofluids, viscoelastic media, and nanoparticle-laden suspensions are often poorly characterized over relevant temperature and field ranges, limiting predictive capability. Surface effects, including zeta potential heterogeneity, roughness-induced field distortions, and thermal slip at coated walls, introduce additional uncertainties that numerical models struggle to capture without high-resolution experimental data. Moreover, most studies focus either on fundamental fluid physics aspects or on application-specific demonstrations, with comparatively fewer works attempting to bridge these scales and propose unified design guidelines that explicitly account for heat transfer under electrokinetic operation. Emerging approaches using machine learning and data-driven modeling to correlate operating conditions, geometry, and thermal performance in complex electrokinetic microflows are just beginning to appear and remain underexplored.

Experimental challenges compound these issues. Resolving temperature and charge-density fields in sub-micron channels demands further advances in noninvasive diagnostics, such as Raman thermometry, quantum-dot-based sensing, or interferometric techniques that overcome limitations of fluorescence and IR methods. Validating models across a wide parameter space, spanning from Newtonian to non-Newtonian fluids, DC to MHz AC fields, and micro- to nano-scale channels, requires standardized benchmark datasets and inter-laboratory comparisons, which are currently scarce [89]. Moreover, scaling from single-channel studies to integrated multi-module lab-on-a-chip systems introduces thermal crosstalk and power management bottlenecks that few studies address holistically.

Validation of models against experiments is a critical step, especially since electrokinetic and thermal effects are highly sensitive to surface properties, channel fabrication, and fluid composition. Benchmark problems, such as electro-osmotic flow with uniform Joule heating in straight channels under well-characterized boundary conditions, serve as testbeds for comparing analytical solutions, numerical simulations, and experimental data, and have been reported in several electrokinetic and ACET studies.

Key challenges in modeling and validation include the following:

- Accurate, temperature-dependent property data for real biofluids, buffers, and nanofluids over the relevant temperature and frequency ranges.
- Capturing surface heterogeneity, random roughness, and dynamic zeta potential in microfabricated channels, which can locally distort fields and heat generation.
- Handling strong coupling and potential instabilities when Joule heating significantly alters conductivity and permittivity, especially at high electric fields.
- Incorporating non-Newtonian and multiphase effects, as in Jeffery fluids, hybrid nanofluids, and blood-like suspensions, which modify both hydrodynamics and thermal transport.

Emerging work is beginning to integrate data-driven approaches and optimization frameworks with high-fidelity simulations, aiming to explore large design spaces and identify electrokinetic microchannel configurations that achieve target thermal and hydraulic performance. As models and measurements become more closely coupled, there is an increasing opportunity to establish standardized datasets and benchmark cases that can accelerate progress toward reliable, thermally aware electrokinetic microfluidic technologies.

Looking forward, several research opportunities promise to overcome these hurdles. Multiphysics models incorporating non-equilibrium EDLs, machine learning-accelerated property estimation, and hybrid continuum–molecular descriptions will enable accurate predictions in extreme regimes, such as high-voltage (>100 V/cm) operation or extreme aspect-ratio channels. Data-driven approaches, trained on coupled experimental–simulation datasets, can surrogate complex nonlinearities to rapidly explore design spaces for thermally optimized devices. Advanced fabrication techniques—3D-printed electrodes, nanocomposite substrates with tailored thermal–electrical properties, and adaptive coatings—offer pathways to engineer thermal gradients for enhanced mixing, pumping, or separation without external controls.

Integration with emerging technologies will further expand impact. In biomedical applications, closed-loop thermal feedback using on-chip sensors and AI controllers can maintain biocompatibility during electroporation or cell sorting, enabling personalized diagnostics. For electronics cooling, hybrid electrokinetic–magnetohydrodynamic or electrokinetic-phase-change systems could handle >500 W/cm^2^ fluxes, supporting next-generation 3D ICs and photonics. Electrified lab-on-disc and wearable platforms stand to benefit from low-power ACET micropumps with multiphase actuation, minimizing battery drain while achieving robust fluid handling. Finally, electrokinetic energy harvesters that exploit temperature–zeta potential coupling could power self-sustaining sensors in IoT and remote diagnostics.

To accelerate progress, the community should prioritize the following:

- Benchmarking: Standardized test cases for electro-osmotic Poiseuille–Couette flows with Joule heating, ACET vortex formation, and conjugate heat transfer across materials.
- Open datasets: Shared high-resolution temperature, velocity, and current measurements for model training and validation.
- Design guidelines: Dimensionless maps correlating operating conditions, geometry, and performance metrics (e.g., Nusselt number, thermal resistance, coefficient of performance) for electrokinetic microdevices.
- Interdisciplinary collaboration: Merging microfluidics expertise with materials science, ML, and systems engineering to tackle multiscale thermal challenges.

By resolving these challenges, heat transfer-aware electrokinetic microfluidics can fully achieve its transformative potential in precision medicine, sustainable cooling, and autonomous platforms, driving innovations aligned with 2030 technology roadmaps.

## 6. Conclusions

This review underscores the importance of thermal effects in microfluidic electrokinetic flow systems that govern flow dynamics, species transport, device reliability, and, ultimately, practical applicability. Because electric-field-driven transport intrinsically couples fluid mechanics, charge transport, and thermal effects, any realistic description of electrokinetic phenomena must explicitly account for internal heat generation, conjugate heat transfer through the surrounding media, and the pronounced temperature dependence of fluid and interfacial properties. Across canonical electro-osmotic flows, AC electrothermal flows, and systems involving non-Newtonian or nanoparticle-laden fluids, a unifying theme emerges: Joule heating and electrothermal effects can either undermine performance or be deliberately exploited as functional mechanisms, depending on the level of thermal control embedded in the design.

The body of literature reviewed in this paper reflects substantial advances in analytical works, high-resolution numerical modeling, and experimental diagnostics, collectively establishing a robust foundation for analyzing coupled electro-thermo-hydrodynamic transport in increasingly complex fluids and geometries. Application-driven studies spanning bioanalytical microchips, electrokinetic microchannel heat sinks, electrified lab-on-disc platforms, and wearable diagnostic systems demonstrate that thermally informed electrokinetic design can yield marked improvements in throughput, resolution, and heat dissipation while maintaining biocompatible and material-safe temperature limits. Nevertheless, key challenges remain, including non-equilibrium electric double-layer behavior under strong electric fields, limited thermophysical property data for realistic biofluids and nanofluids, and the difficulty of predicting thermal crosstalk in densely integrated microsystems.

Future progress is likely to be driven by the integration of first-principles modeling with data-driven methods and systematic optimization, supported by standardized benchmark problems and high-quality open datasets. Such approaches will facilitate efficient exploration of expansive design spaces encompassing multiphase AC electrothermal pumping, hybrid electro-osmotic–pressure-driven cooling strategies, and devices employing advanced substrate and coating materials engineered for simultaneous electrical and thermal performance. By prioritizing heat transfer as a primary key contributor in such systems, the thermal management and microfluidics communities can advance toward robust, scalable, and energy-efficient platforms for precision diagnostics, high-heat-flux removal applications, and autonomous microsystems.

## Data Availability

No new data was created in this research.

## Abbreviations

The following abbreviations are used in this manuscript:

ACET	AC electrothermal
CFD	Computational Fluid Dynamics
EDL	Electric double layer
EOF	Electro-osmotic flow
NS	Navier–Stokes
PNP	Poisson–Nernst–Planck
